# TiO_2_-Coated Silicon Nanoparticle Core-Shell Structure for High-Capacity Lithium-Ion Battery Anode Materials

**DOI:** 10.3390/nano13071144

**Published:** 2023-03-23

**Authors:** Jinbao Li, Sha Fan, Huijuan Xiu, Haiwei Wu, Shaoyan Huang, Simin Wang, Dingwen Yin, Zili Deng, Chuanyin Xiong

**Affiliations:** College of Bioresources Chemical & Materials Engineering, Shaanxi University of Science & Technology, Xi’an 710021, China

**Keywords:** lithium-ion batteries, silicon-based anode materials, SiNPs@TiO_2_ core-shell structure, AgNWs doping

## Abstract

Silicon-based anode materials are considered one of the highly promising anode materials due to their high theoretical energy density; however, problems such as volume effects and solid electrolyte interface film (SEI) instability limit the practical applications. Herein, silicon nanoparticles (SiNPs) are used as the nucleus and anatase titanium dioxide (TiO_2_) is used as the buffer layer to form a core-shell structure to adapt to the volume change of the silicon-based material and improve the overall interfacial stability of the electrode. In addition, silver nanowires (AgNWs) doping makes it possible to form a conductive network structure to improve the conductivity of the material. We used the core-shell structure SiNPs@TiO_2_/AgNWs composite as an anode material for high-efficiency Li-ion batteries. Compared with the pure SiNPs electrode, the SiNPs@TiO_2_/AgNWs electrode exhibits excellent electrochemical performance with a first discharge specific capacity of 3524.2 mAh·g^−1^ at a current density of 400 mA·g^−1^, which provides a new idea for the preparation of silicon-based anode materials for high-performance lithium-ion batteries.

## 1. Introduction

With the increasing human demand for energy, the development of a green economy of energy has become an inevitable trend. Clean energy sources such as solar, wind, and geothermal energy are limited by conditions that prevent them from being used directly, requiring them to be converted and stored. Electrochemical energy storage technology can convert and store energy efficiently, which plays an important role in the development of green energy. Among the many electrochemical energy storage technologies, lithium-ion batteries are widely used in mobile electronic devices due to their excellent self-discharge performance and portability, showing a broad application prospect [1]. In addition, supercapacitors [2,3,4] and zinc-air batteries [5], which have high energy density and excellent safety, have also received wide attention from researchers. Since the capacity of conventional graphite electrodes of lithium-ion batteries is close to the theoretical maximum, it cannot meet the demand. Therefore, there is a need to develop higher-capacity battery cathode materials [6]. Silicon-based materials are considered one high-capacity anode material with great potential for development because of their higher theoretical capacity and lower embedded lithium potential compared with conventional graphite electrodes and abundant natural resources [7,8,9]. However, silicon cathode materials also have the following disadvantages: silicon undergoes volume changes during charging and discharging, resulting in stress-strain and leading to cracks or even pulverization of the silicon cathode; the solid electrolyte interface film (SEI) is unstable, and the silicon cathode material after cracking exposes a fresh surface which can form an SEI film again and hinder the migration of Li^+^ [10]; the structural changes and volume effects of the silicon cathode lead to the cracking of the silicon cathode during repeated lithiation process of silicon cathode material from the electrode structure, resulting in the rapid failure of the battery [11,12,13]. To address these problems, current research has focused on both silicon nanosizing [14,15] and designing silicon-based composites to mitigate the bulk expansion of silicon and maintain the stability of SEI films.

Nanotechnology enables changes in the physical and chemical properties of materials as an effective way to improve anode materials for lithium-ion batteries. It has been found that after nanosizing the size structure of silicon, the high specific surface area shortens the diffusion distance of lithium ions in the process of lithium removal and embedding; Furthermore, it is relatively less stressed and the chalking phenomenon is significantly reduced. Tang et al. [16] used graphene oxide nanosheets as a template to synthesize silicon nanosheets (Si-NSs) with Si-NSs size of 10 nm, which were used as anode materials for lithium-ion batteries. The battery capacity was maintained at 800 mAh·g^−1^ after cycling. Nanosizing the structure of silicon has a high specific surface area, which can make sufficient contact between the material and electrolyte, thereby improving electrochemical performance. Although the nanosized silicon cathode can improve the battery performance, the surface of some silicon nanomaterials is still insufficient to avoid a large number of crushing phenomena. Carbon materials do not have much volume change during charging and discharging and have high electrical conductivity. To further improve the battery performance, carbon materials and silicon particles have been prepared as composites [17,18].

Yang et al. [19] obtained porous silicon nanosheets by etching an iron-silicon alloy and preparing composites from artificial graphite and silicon. The specific capacity was 445 mAh·g^−1^ at a current density of 0.5 A·g^−1^. Park et al. [20] prepared a 10 nm carbon coating on Si nanosheets using chemical vapor deposition (CVD). It showed promising electrochemical properties at a current density of 0.15 C. Alternatively, when a layer of carbon material is coated on the surface of the Si nanoparticles to form a core-shell structure, carbon material can be used as a bulk buffer layer for the Si nanosheets to prevent the electrolyte from participating in the reaction with the electrode, which reduces the formation of SEI film and keeps the SEI film stable. This reduces the reversible capacity loss of Li-ion batteries. Ma et al. [21] prepared (Si/PC) nanoparticles by coating silicon with porous carbon (PC), and the first discharge specific capacity was 1586.3 mAh·g^−1^ when the mass ratio of Si/PC nanoparticles to graphite was 2:1. Su et al. [22] synthesized Al-Si/RF with core-shell structure using resorcinol formaldehyde (RF) as the carbon source and Al-Si alloy spheres as the Si source. After an etching by HCl, the Si/C shell layer was obtained by pyrolysis at 800 °C. The results show that the capacity retention of the Si/C shell layer after cycling is 63.05%. It indicates that the Si/C shell layer has enough space to buffer the volume contraction/expansion of silicon and prevent the severe pulverization of the electrode during the alloy reaction. However, the Si-based anode generates a large amount of heat during cycling and the carbon material shell layer still has undesirable exothermic behavior with a risk of explosion [23]. Metals have excellent electronic conductivity and mechanical properties to achieve better energy storage performance [2,5], providing a way to improve the electrochemical performance of silicon-based lithium-ion battery anodes. Yin et al. [24] homogeneously combined a small number of silver nanoparticles into silicon-carbon Si/C composites to improve the electrochemical performance of silicon-based lithium-ion battery anodes. Compared with Si/C anodes, Si/Ag/ C nanohybrids exhibited excellent multiplicative performance and the presence of silver nanoparticles also contributed to improved cycling stability, suggesting that metal nanoparticles can increase charge carrier mobility and improve the electrochemical performance of silicon-based lithium-ion battery anodes. Zhang et al. [25] decorated metal nanocrystals (Ag, Cu, and Fe) on the surface of polydopamine-derived carbon-coated commercial silicon nanoparticles. The results showed that the metal nanocrystals reduced interparticle resistance promoted the diffusion of lithium ions and also reduced lithium-ion consumption, exhibiting significant multiplicity and cycling performance when used as anode materials for batteries.

Anatase (TiO_2_) has been widely used in lithium-ion batteries because of its excellent chemical stability, relatively high ionic conductivity, and low bulk effect during charging and discharging [26,27,28]. Li et al. [29] used the sol-gel method and thermal treatment process to prepare nanoparticles with core-shell structure (Si/TiO_2_). Compared with pure SiNPs, the Si/TiO_2_ electrode exhibited excellent electrochemical performance with an initial capacity of up to 2636.9 mAh·g^−1^. Chen et al. [30] used a dual protection strategy to embed Si nanoparticles into spherical carbon (s-C) by hydrothermal method and coated the outer surface with a TiO_2_ shell to obtain Si/s-C@TiO_2_ composites. With the buffering of the bulk effect of silicon nanoparticles by spherical carbon and the TiO_2_ shell with high mechanical strength on the surface, the Si/s-C@TiO_2_ composites exhibit excellent electrochemical properties. However, the lower Li^+^ diffusion coefficient (~10–17 cm^2^·S^−1^) [31] and poor electrical conductivity (~10–12 S·cm^−1^) [32] of TiO_2_ itself affect the application in Li-ion batteries.

**Figure 1:**
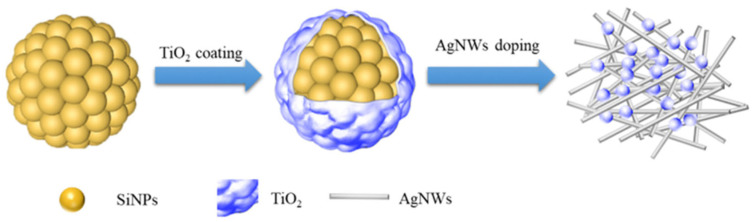
Schematic structure of SiNPs@TiO_2_/AgNWs.

To achieve composites with high conductivity, this work proposes to form a core-shell structure with silicon nanoparticles (SiNPs) as the nucleus and anatase TiO_2_ coating. The TiO_2_ shell layer provides mechanical support which both alleviates the bulk effect and stabilizes the SEI film, and reduces the direct contact between the electrolyte and the active material; the repetitive generation of SEI film is therefore reduced and the capacity of the battery is improved. The doping of AgNWs improves the electrical conductivity of the material. The SiNPs@TiO_2_/AgNWs anode material with a conductive network structure is prepared as the electrode material for Li-ion batteries, whose structure is schematically shown in Figure 1. The synergistic effect of the core-shell structure and the conductive network structure to enhance the cycling stability of the silicon-based anode provides a new idea for the preparation of silicon-based anode materials for high-performance lithium-ion batteries.

## 2. Materials and Methods

### 2.1. Chemicals and Materials

Silicon nanoparticles (SiNPs, 99.9%) were purchased from Chaowei Nanotechnology Co., Ltd., Shanghai, China. Hydrochloric acid (HCl), tetra butyl titanate (TBT), N-methyl pyrrolidone (NMP), silver nitrate (AgNO_3_), and anhydrous ethanol were purchased from Damao Chemical Reagent Factory, Tianjin, China. Polyvinylpyrrolidone (PVP) and sodium bromide (NaBr) were purchased from Aladdin Biochemical Technology Co., Ltd., Shanghai, China. Ethylene glycol (EG) was purchased from Kolon Chemical Co., Ltd., Chengdu, China. Conductive carbon black (CB) was purchased from Kejing Material Technology Co., Ltd., Hefei, China. Polyvinyl alcohol (PVA, 99.9%) was purchased from Urso Chemical Technology Co., Ltd., Jingnan, China.

### 2.2. Preparation of SiNPs@TiO_2_/AgNWs anode Materials

Preparation of SiNPs@TiO_2_ composites: 0.3 g of SiNPs was first added to 3.5 mL of anhydrous ethanol and 0.5 mL of deionized water and dispersed well by sonication, which was recorded as solution A. Then, anhydrous ethanol, 0.4 mL of HCl, and 2.5 mL of TBT were mixed and stirred for 20 min, which was recorded as solution B. Then, solution A was added to solution B drop by drop and stirred for 4 h. Finally, it was dried at 65 °C and put into a muffle furnace, and calcined at 500 °C for 2 h to obtain SiNPs@TiO_2_.

**Figure 2:**
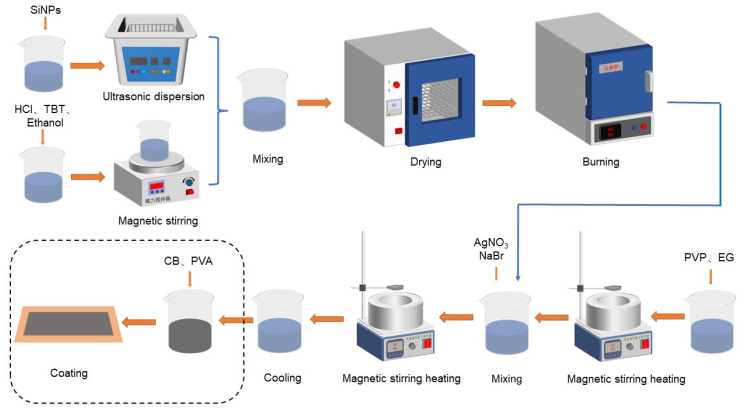
Schematic diagram of the preparation process.

Preparation of SiNPs@TiO_2_@AgNWs negative electrode material: the AgNWs was prepared by referring to the polyol method in the literature [33]. First, 0.4 g PVP and 50 mL EG solution were mixed and heated by magnetic stirring, then 0.15 g SiNPs@TiO_2_ was added to the above solution and stirred to react for 1 h. AgNO_3_ and NaBr were added to the above reaction and continued to be stirred and heated for 1 h. Finally, after standing and cooling to room temperature, solid particles were obtained after washing with anhydrous ethanol and water (as shown in Figure 2).

The SiNPs@TiO_2_/AgNWs, conductive carbon black, and binder (PVA) were dried in a vacuum drying oven for 12 h at a temperature of 80 °C to remove the moisture from the materials. The copper foil material is soft, relatively stable in air, and does not develop in dry air; therefore, copper foil is used as the negative collector fluid. The copper foil was cut to a certain size and pasted onto the glass plate, wiped with anhydrous ethanol to remove surface dirt, and dried for use. Finally, SiNPs@TiO_2_/AgNWs, conductive carbon black, and binder (PVA) were mixed well with a mass ratio of 6:2:2, applied on the copper foil with a squeegee, and dried under vacuum at 60 °C for 12 h.

### 2.3. Material Structure Characterization

The surface morphology of the samples was observed by scanning electron microscopy (S4800, Nippon Institute of Science Co., Ltd., Beijing, China). Transmission electron microscopy (G2F20 S-TWIN, FEI America, Inc., Shanghai, China) was used to observe the microstructure of the core-shell material. An X-ray diffractometer (D8 Advance, Nippon Institute of Science Co., Ltd., Beijing, China) was used to examine the crystal features of the samples.

### 2.4. Electrochemical Measurement

The dried electrode sheets were cut to 16 mm and lithium sheets were used as counter electrodes. The cells were assembled in a glove box according to the sequence [34]. The electrolyte was 1 mol L^−1^ LiPF_6_ (VEC: VDMC = 1:1) with a commercial polypropylene diaphragm.

The electrochemical properties were tested on a LAND cell test system (CT 3001A, Blue Electric Electronics Co., Ltd., Wuhan, China) in the room temperature voltage range of 0.01–2 V. Cyclic voltammetry (CV) and electrochemical impedance spectroscopy (EIS) were performed on an electrochemical workstation (P4000+, Princeton, NJ, USA) with a scan rate of 0.02 mVs−1 for CV and a frequency range between 100 MHz and 100 KHz for EIS.

## 3. Results and Discussion

**Figure 3:**
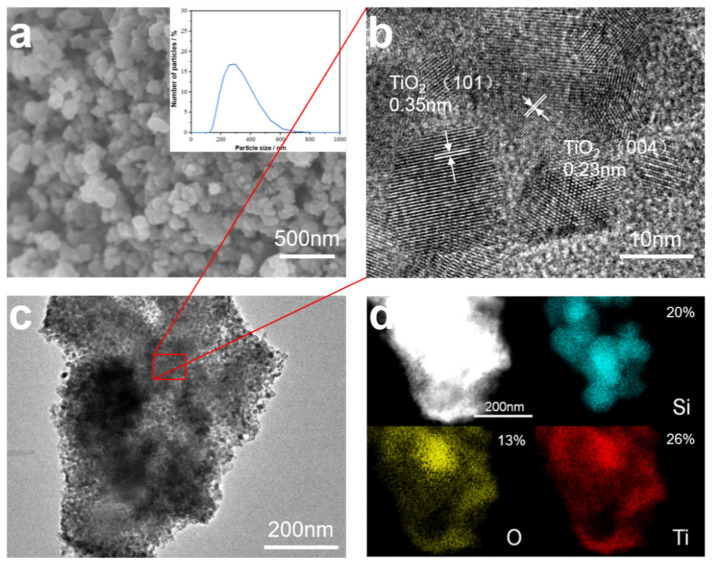
(**a**) SEM image of SiNPs@TiO_2_, whose upper right inset shows the particle size distribution of SiNPs@TiO_2_, (**b**) HRTEM image, (**c**) TEM image, and (**d**) EDS image.

To confirm the bonding state of SiNPs and TiO_2_, the surface morphology and elemental characterization were analyzed. The SEM image of the SiNPs@TiO_2_ composite is shown in Figure 3a, from which it can be observed that the surface of the material is mostly a irregular spherical structure and the overall distribution of particle size is more uniform with an average particle size of 257.3 nm. Figure 3b shows the HRTEM image, which shows that nanoparticles have clear lattice stripes, and the crystalline surface spacing is 0.35 nm as measured by software, which is typical of the (101) crystalline surface of anatase TiO_2_ [35]. The structure of the composites was further observed by transmission electron microscopy as shown in Figure 3c, and the overall SiNPs@TiO_2_ showed an encapsulated structure with many small particles encapsulated on the surface of SiNPs. Combined with the EDS analysis pattern in Figure 3d, the distribution regions of Si, O, and Ti elements can be observed, which further proves that TiO_2_ is encapsulated with SiNPs, indicating the successful preparation of SiNPs@TiO_2_ composites with core-shell structure.

**Figure 4:**
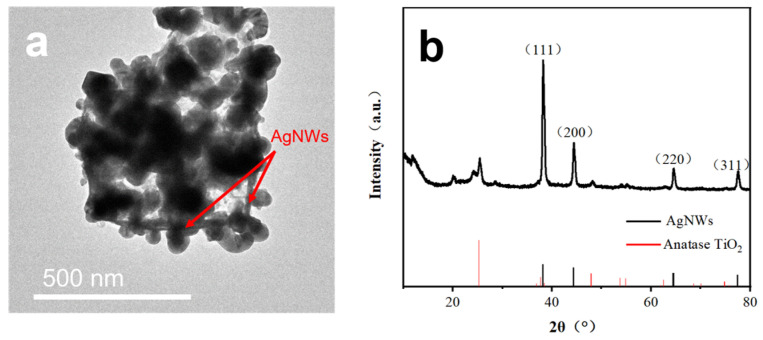
SiNPs@TiO_2_/AgNWs (**a**) TEM image, and (**b**) XRD spectrum.

Since the conductivity of TiO_2_ is low, AgNWs was introduced to improve the conductivity of the electrode. The TEM of SiNPs@TiO_2_/AgNWs is shown in Figure 4a, and linear AgNWs doping can be observed in the SiNPs@TiO_2_ material, indicating the successful introduction of AgNWs into the SiNPs@TiO_2_ composite. From the XRD pattern of the SiNPs@TiO_2_/AgNWs composite in Figure 4b, it can be observed that four clear diffraction peaks of AgNWs appear at 38.1°, 44.2°, 64.4°, and 77.4°, which compare to the results with standard PDF cards, respectively, corresponding to the (111), (200), (220), and (311) face-centered cubic phase silver crystal plane diffraction [36]. Among them, the (111) face diffraction peak of AgNWs is the strongest, indicating that AgNWs is preferentially oriented crystalline growth along the (111) direction. Meanwhile, there are no obvious impurity diffraction peaks, indicating that the prepared products are of high purity and have a face-centered cubic crystal structure.

**Figure 5:**
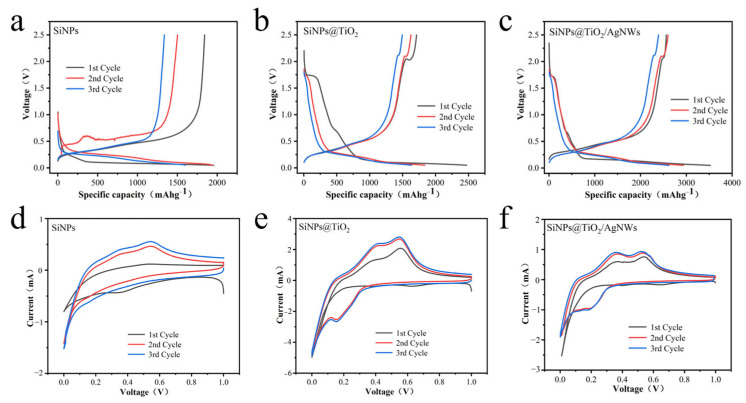
Charge/discharge curves and cyclic voltammetry curves of different anode materials (**a**,**d**) SiNPs, (**b**,**e**) SiNPs@TiO_2_, and (**c**,**f**) SiNPs@TiO_2_/AgNWs.

To test the electrochemical performance of this cathode material, the battery assembled with it was subjected to constant current charge and discharge tests. Figure 5a–c shows the charge/discharge curves of SiNPs, SiNPs@TiO_2_, and SiNPs@TiO_2_/AgNWs at a current density of 400 mA·g^−1^. From Figure 5a, the first charge/discharge specific capacities of 1842.3 mAh·g^−1^ and 1955.8 mAh·g^−1^ and the Coulomb efficiency of 94.19% were obtained when SiNPs were used as the anode material of the battery. There is a large interval between the voltage and specific capacity curves of the batteries in the subsequent cycles, indicating that the specific capacity loss of the batteries is large. Figure 5b shows the charge and discharge curves of SiNPs@TiO_2_ as the anode material, the first charge-specific capacity is 1711.9 mAh·g^−1^ and the discharge-specific capacity reaches 2472.5 mAh·g^−1^ with a coulomb efficiency of 69.24%. The specific capacity of the batteries is improved compared with SiNPs, and the curve overlap is relatively good in the subsequent cycles, indicating that the specific capacity loss of the batteries is small. Figure 5c shows the charging and discharging curves of SiNPs@TiO_2_/AgNWs as anode materials, and the first charging specific capacity and discharging specific capacity are 2561.772 mAh·g^−1^ and 3524.242 mAh·g^−1^. The coulomb efficiency is maintained at 72.69%. Compared with SiNPs@TiO_2_ anode material, the specific capacity was significantly improved and the Coulomb efficiency was also enhanced. Meanwhile, the good overlap of voltage and specific capacity curves in the next cycle and the cycle stability of the cell were improved, indicating that the addition of AgNWs improved the electronic conductivity of the electrode [2,5] and the utilization of the active material was improved, thus enhancing the specific capacity of the batteries.

Cyclic voltammetry tests were performed on the batteries to investigate the redox reactions occurring on the electrodes during charging and discharging. Figure 5d–f shows the cyclic voltammetry curves of SiNPs, SiNPs@TiO_2_, and SiNPs@TiO_2_/AgNWs negative electrode materials for three cycles. It can be observed from the figures that the three groups of SiNPs, SiNPs@TiO_2_, and SiNPs@TiO_2_/AgNWs materials show a reduction peak between 0.3–0.7 V in the first cycle and disappear in the subsequent cycles, which is due to the reaction between the electrolyte and active material on the electrode surface to generate SEI film. The silicon negative electrode is highly susceptible to breakage and exposure during lithium ion embedding, thus repeated forming of SEI film and consuming of excessive lithium ions resulted in capacity loss. From Figure 5d, it can be observed that there is a large interval between the curves of SiNPs in the subsequent cycles, indicating a large loss of specific capacity of the cell. In the second cycle of SiNPs@TiO_2_ in Figure 5e, the reduction peak between 0.1–0.3 V represents the formation of silicon-lithium alloy (LixSi). The oxidation peak is between 0.3–0.7 V, which represents the removal and embedding of lithium elements from the silicon-lithium alloy [37]. Moreover, the curve overlap is relatively good in the subsequent cycles, showing a decrease in the specific capacity decay of the batteries. It indicates that the core-shell structure formed by TiO_2_ as the cladding layer well encapsulates the SiNPs, which reduces the decay of specific capacity to some extent. Among the three groups of anode materials, the curve overlap of SiNPs@TiO_2_/AgNWs is the best, as shown in Figure 5f; the curve shift is relatively small, which means the cycle stability of the battery is better. Due to the doping of AgNWs again based on TiO_2_ coating to form a conductive network structure and improve the conductivity of the material, the specific capacity of the prepared SiNPs@TiO_2_/AgNWs anode material is improved.

**Figure 6:**
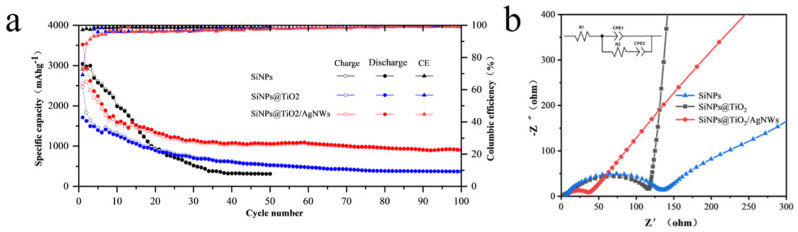
SiNPs, SiNPs@TiO_2_ and SiNPs@TiO_2_/AgNWs (**a**) cyclic characteristic curves, and (**b**) EIS, the illustration in the upper left corner shows the equivalent circuit.

The cyclic charge/discharge test was performed at a high current density of 1 A·g^−1^ to test the cyclic stability of the battery. The cyclic performance of the three groups of SiNPs, SiNPs@TiO_2_, and SiNPs@TiO_2_/AgNWs materials is shown in Figure 6a. After 50 charge/discharge cycles, the discharge-specific capacity was 310 mAh·g^−1^, which was high in the initial discharge-specific capacity but decayed rapidly during the subsequent cycles. The initial discharge specific capacity of SiNPs@TiO_2_ and SiNPs@TiO_2_/AgNWs were 2472.5 mAh·g^−1^, 3524.2 mAh·g^−1^, the discharge specific capacity was reduced to 372.77 mAh·g^−1^, 1157.6 mAh·g^−1^. After 100 cycles, the capacity retention rate was 15%, 32.83%. Compared with SiNPs@TiO_2_ anode material, SiNPs@TiO_2_/AgNWs anode material has better cycling stability; furthermore, the specific capacity of SiNPs@TiO_2_/AgNWs anode material is increased. Compared to titanium dioxide coated silicon nanoparticles (SiNPs@TiO_2_) [29], the doping of AgNWs resulted in higher cycling stability and capacity retention. Meanwhile, higher first discharge specific capacity was observed compared to the silicon coated silver nanowires (AgNWs@Si) composite [38]. It indicates that the decrease in the specific capacity of the battery can be alleviated to some extent after doping with AgNWs. On the one hand, due to the excellent electrical conductivity of AgNWs, the electron conduction rate is improved and the utilization rate of the active material is increased, thus improving the specific capacity of the battery; on the other hand, combined with the SEM images, AgNWs has a high aspect ratio and build a stable conductive network, which strengthens the internal structure of the material and helps to disperse the stress inside the material, thus the stability of the electrode is improved.

To further investigate the charge transfer kinetics of the batteries, the electrochemical impedance spectra (EIS) of SiNPs, SiNPs@TiO_2_, and SiNPs@TiO_2_/AgNWs anode materials were analyzed. The semicircle in the high-frequency region of the EIS indicates the reaction between the electrode material and the electrolyte such that the capacitance generated by the bilayer interacts with Li^+^ to form an exchange resistance. The slope of the low-frequency region represents the degree of solid diffusion of lithium ions inside the active material particles, also known as Warburg diffusion impedance. From Figure 6b, it can be seen that the overall trend of the EIS curves of the three materials is similar. The SiNPs have the largest semicircle diameter, which is mainly due to the volume contraction and expansion of silicon during the process of lithium de-embedding, which causes the rupture of the material and makes the silicon material continuously expose new surfaces, which react with the electrolyte to generate a new SEI film, making the charge transfer resistance in the material increase. Compared with SiNPs, the semicircular diameter of SiNPs@TiO_2_ decreases and the charge transfer resistance decreases, indicating that after TiO_2_ coating, the bulk effect on the material is alleviated and the chalking phenomenon of silicon material and the repetitive formation of SEI film are reduced; therefore, the charge transfer resistance decreases compared with SiNPs. The semicircular diameter of SiNPs@TiO_2_@AgNWs is the smallest. The charge transfer resistance of the electrode is significantly lower, which indicates that the overall conductivity of the electrode material is improved after AgNWs doping. The slope of both SiNPs@TiO_2_ and SiNPs@TiO_2_/AgNWs is larger than that of SiNPs and has a higher lithium-ion diffusion coefficient [39]. The larger slope of the SiNPs@TiO_2_ anode material with low lithium-ion diffusion impedance is caused by the TiO_2_ layer on the surface of the silicon nanoparticles, the structure of which improves the migration of lithium ions to the surface of the silicon nanoparticles [40]. After doping with AgNWs, the electronic conductivity inside the electrode is improved [2,5], the charge transfer impedance is reduced, and the slope of the SiNPs@TiO_2_/AgNWs anode material in the low frequency region, i.e., the Warburg impedance Zw is reduced, indicating that the ion diffusion rate inside the battery decreases after the addition of AgNWs. This may be attributed to the use of AgNWs, as a silicon-based lithium-ion battery Zw values are smaller when AgNWs is used as an anode material for silicon-based Li-ion batteries [38].

**Figure 7:**
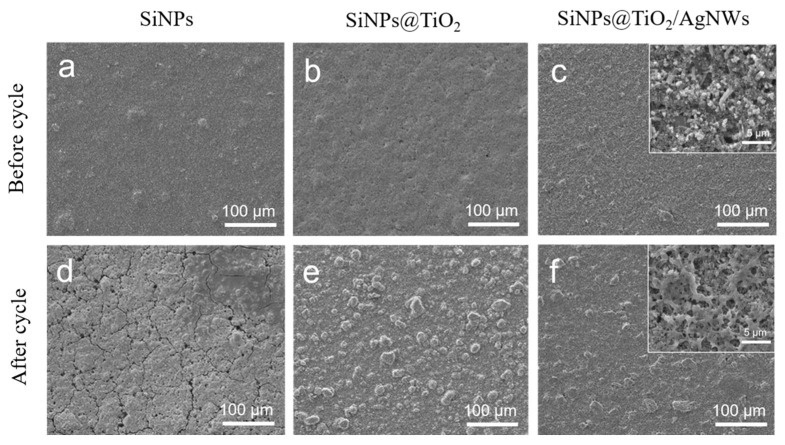
SEM images of the surface of different battery cathode materials before and after 100 cycles of charging and discharging (**a**,**d**) SiNPs, (**b**,**e**) SiNPs@TiO_2_, and (**c**,**f**) SiNPs@TiO_2_/AgNWs.

To further investigate the effect of structure on the electrochemical performance of the battery, the surface of the battery electrode sheet was characterized morphologically. From Figure 7a, it can be observed that the electrode surface of the SiNPs negative electrode sheet is flat before cycling, after cycling, it can be observed by Figure 7d that there are obvious cracks on the surface of the electrode sheet, which is caused by the volume expansion and the situation is serious enough to cause particle shedding. The surface of the SiNPs@TiO_2_ negative electrode sheet after TiO_2_ coating is flatter as shown in Figure 7b, after cycling, more obvious granular material can be seen in Figure 7e, but there is no fine crack, which indicates that the TiO_2_ shell layer has a certain buffering effect on the volume expansion of the active material, and it reduces the further reaction between the electrolyte and the active material and stops the excessive consumption of lithium ions. After doping with AgNWs as shown in Figure 7c, the overall homogeneity of the SiNPs@TiO_2_/AgNWs material is improved, and the material structure remains intact after cycling, as can be observed from Figure 7f. Under high magnification, it can be observed that the surface of the electrode sheet shows a mesh structure, and this mesh structure enhances the stability of the active material and maintains the structural integrity of the electrode sheet, which allows the electrode to be stable during the subsequent charging and discharging cycles.

## 4. Conclusions

In this work, we address the problems of volume effect and instability of solid electrolyte interface film (SEI) in the silicon cathode of a Li-ion battery and form a core-shell structure by encapsulating SiNPs with TiO_2_. TiO_2_ acts as a buffer layer, which reduces the direct contact between electrolyte and active material and inhibits the repeated formation of SEI film while alleviating the volume change of silicon-based material and avoiding the pulverization of silicon. Moreover, the introduction of AgNWs enabled the formation of a conductive network structure, which not only enhanced the conductivity of the material but also alleviated the shedding of the active material from the collector fluid. The results of SiNPs@TiO_2_/AgNWs composites as anode materials for Li-ion batteries showed that the material exhibited good electrochemical performance through the synergistic effect of the core-shell structure and the conductive network structure, with 400 mA·g^−1^ The first discharge-specific capacity at current density reaches 3524.2 mAh·g^−1^, which is 80.2% higher than that of pure SiNPs electrode, indicating that the core-shell structure design can effectively mitigate the bulk effect and provide a new idea for the preparation of silicon-based anode materials for high-performance lithium-ion batteries.

## Data Availability

The data presented in this study are available on request from the corresponding author.
